# One-Pot CRISPR-Cas12a-Based Viral DNA Detection via HRP-Enriched Extended ssDNA-Modified Au@Fe_3_O_4_ Nanoparticles

**DOI:** 10.3390/bios14010026

**Published:** 2024-01-02

**Authors:** Dong Hyeok Park, Izzati Haizan, Min Ju Ahn, Min Yu Choi, Min Jung Kim, Jin-Ha Choi

**Affiliations:** 1 School of Chemical Engineering, Clean Energy Research Center, Jeonbuk National University, 567 Baekje-daero, Deokjin-gu, Jeonju-si 54896, Jeollabuk-do, Republic of Korea; 201610776@jbnu.ac.kr (D.H.P.); minyu5089@jbnu.ac.kr (M.Y.C.); minjeong413@jbnu.ac.kr (M.J.K.); 2 Department of Bioprocess Engineering, Jeonbuk National University, 567 Baekje-daero, Deokjin-gu, Jeonju-si 54896, Jeollabuk-do, Republic of Korea; izzatihaizan22@jbnu.ac.kr; 3 Department of Biotechnology, Jeonbuk National University, 79 Gobongro, Iksan-si 54596, Jeollabuk-do, Republic of Korea; ahnminju@jbnu.ac.kr

**Keywords:** signal amplification, Au@Fe_3_O_4_ nanoparticle, CRISPR biosensor, early diagnosis, point-of-care testing

## Abstract

In the context of virus outbreaks, the need for early and accurate diagnosis has become increasingly urgent. In addition to being crucial for effective disease control, timely and precise detection of viral infections is also necessary for the implementation of essential public health measures, especially during pandemics. Among these measures, point-of-care testing (POCT) stands out as a powerful approach with the potential to revolutionize the landscape of viral diagnosis. In this study, we developed a one-pot clustered regularly interspaced short palindromic repeats (CRISPR)-Cas12a-based viral DNA detection system tailored for POCT; this method utilizes multi-enzyme-modified Au@Fe_3_O_4_ nanoparticles. As an alternative to nucleic acid amplification, our method uses single-stranded DNA elongation to facilitate multi-enzyme modification; this guarantees heightened sensitivity and expedites the diagnostic process. We achieved a satisfactory limit of detection of 0.25 nM, demonstrating the remarkable sensitivity of the method without the need for sophisticated equipment. The incorporation of Au@Fe_3_O_4_ magnetic nanoparticles facilitates sample separation, further streamlining the workflow and reinforcing the simplicity of our method. This integrated approach offers a practical solution for sensitive viral DNA detection in POCT scenarios, advancing the field of rapid and accurate diagnostics.

## 1. Introduction

Cervical cancer is a significant global health issue, ranking as the fourth most common cancer and the fourth leading cause of cancer-related deaths among women worldwide [1,2,3,4]. It is estimated that about 80% of women will contract human papillomavirus (HPV) in their lifetime, but the majority (~90%) naturally clear the infection within 9–12 months through their innate immunity [5,6,7]. HPV16/18, which are high-risk HPV types, are the most aggressive and persistent [8,9,10]. They integrate with the host genome and cause cervical neoplasia, and they are two of the most significant HPV types linked to reproductive system cancers, accounting for a substantial portion (up to 50%) of cervical cancer cases. Moreover, they are also associated with other forms of reproductive system cancers, similar to different HPV strains [11].

In May 2018, the WHO Director-General initiated a call to action to eliminate cervical cancer; this led to the development of a global strategy in 2019 [12]. The ambitious goals for 2030 included attaining 90% HPV vaccination coverage, ensuring that 70% of women are screened twice in their lifetime, and providing 90% of women access to treatment and palliative care for cervical pre-cancer and cancer [13,14]. While traditional cytology-based screening methods had limitations including sensitivity and the need for frequent screenings, the advent of HPV testing and vaccines, which are more sensitive but slightly less specific, marked a significant advancement [15,16]. Even with this advancement, the implementation of HPV-based screening is still limited, especially in low/middle-income countries (LMICs), where cervical cancer patients face challenges. These limitations include the cost of the tests, the need for laboratory infrastructure, and processing time, which hinder the feasibility of a single-visit approach (SVA) [17].

Therefore, for widespread adoption of primary HPV screening in LMICs, there is a critical need for affordable and POCT HPV tests to enable an SVA. This will aid in achieving the WHO strategic targets for cervical cancer elimination by 2030 [18,19,20,21]. Effective disease control heavily depends on the timely and accurate identification of pathogens. Early virus detection is crucial for the implementation of vital public health measures such as contact tracing, isolation, and quarantine, which are instrumental in curbing further transmission [22,23]. POCT has emerged as a critical approach in this regard. By enabling rapid diagnostic results at a patient’s bedside, POCT facilitates prompt decisionmaking, intervention, and containment efforts [22,24]. The POCT adaptable enzyme-linked immunosorbent assay (ELISA) is a representative colorimetric biosensor for antibody measurements; however, it falls short in sensitivity [25,26]. This limitation leads to inadequate sensitivity for early diagnosis, thus making it unsuitable for early disease detection. While polymerase chain reaction (PCR) offers high sensitivity in detecting viral DNA and RNA, it is plagued by time-consuming procedures, high costs, and the potential for false positive signals [27,28]. These drawbacks hinder its utility in POCT scenarios. Hence, there is an urgent demand for POCT systems that are both portable and easy to implement. Among the POCT systems available, the one-pot technique uses a single-tube configuration, making it suitable for use in LMICs. To eliminate the risk of cross-contamination and the need for separate pre- and post-amplification workspaces, an additional layer of enhanced specificity can be achieved by conducting both the amplification and CRISPR/Cas-based cleavage in a single tube [29,30].

CRISPR-based biosensors have garnered significant attention as a promising approach for precise nucleic acid detection [31]. By utilizing the versatile CRISPR-Cas system, which is renowned for its genome editing capabilities, these biosensors excel in achieving both specificity and sensitivity in detecting target DNA or RNA sequences [32,33]. The CRISPR-Cas12a variant of the CRISPR-Cas family possesses distinct trans-cleavage (collateral) abilities, which enable it to nonspecifically cut nearby single-stranded DNA (ssDNA) upon activation using its corresponding target DNA [34,35,36]. This unique feature has been increasingly utilized in the creation of innovative biosensing methods. Through engineering, detectable signals, including fluorescence or color changes upon binding to the target sequence, can be created. This feature offers simplified detection processes with visual or automated readout options. However, when considering their integration into POCT scenarios, it is difficult for the CRISPR biosensors to streamline the assay while maintaining sensitivity and specificity. The need for intricate sample preparation, nucleic acid extraction, and amplification steps can impede the seamless incorporation of CRISPR biosensors into portable and rapid testing platforms [37,38,39].

**Figure 1:**
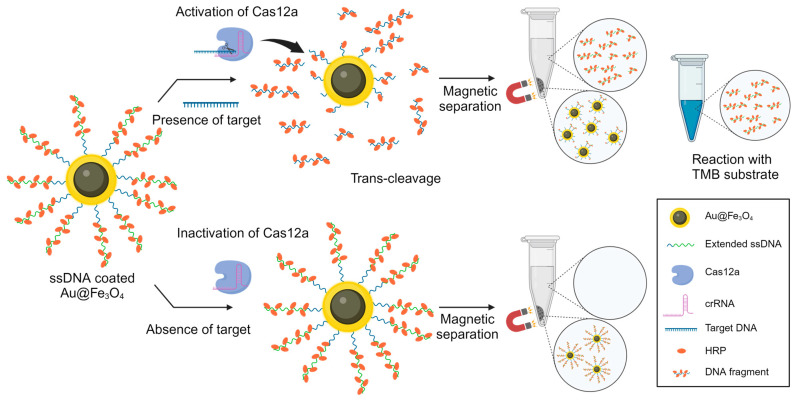
Schematic diagram illustrating the strategy based on target presence for CRISPR-Cas12a-based one-pot detection.

To address these challenges, we propose the development of a system that guarantees simplicity and heightened sensitivity for POCT applications. In this study, we developed a novel CRISPR-based DNA biosensor that harnesses the power of magnetic enzyme complexes within a colorimetric framework to achieve highly sensitive viral DNA detection (Figure 1). Here, we refer to the Au@Fe_3_O_4_ magnetic nanoparticles as gold nanohybrids (AuNHs). This terminology is adopted to succinctly encapsulate their hybrid nature, combining the unique properties of gold (Au) nanoparticles with the magnetic capabilities of iron oxide (Fe_3_O_4_) [40]. The AuNHs were crafted using a magnetic core to facilitate easy separation through magnetism and an outer Au shell to enhance their strong binding affinity with ssDNA. Multiple horseradish peroxidase (HRP)-modified ssDNAs were immobilized on the AuNHs to facilitate signal amplification [41]. To further enhance the signal, we incorporated biotin-dUTP into the ssDNA elongation process; this enhanced streptavidin-HRP binding [42]. Utilizing the trans-cleavage reaction of CRISPR-Cas12a, the attached ssDNAs on the AuNHs underwent enzymatic degradation, releasing multiple HRPs. This resulted in a substantial HRP presence in the supernatant and led to an observable color change during the 3,3′,5,5′-tetramethylbenzidine (TMB) reaction. This color change, which was due to the oxidation of the TMB substrate during the HRP-mediated H_2_O_2_ reduction, visually indicates the presence of the target viral DNA. Our approach capitalizes on the inherent specificity and programmability of the CRISPR-Cas12a system to achieve sensitive detection without traditional nucleic acid amplification steps. By eliminating the need for nucleic acid amplification and capitalizing on signal amplification through ssDNA elongation, our one-pot system achieves both heightened sensitivity and significant time efficiency. Notably, the use of AuNHs facilitates easy separation within 5 min through magnetism, thus eliminating the need for specialized equipment, such as centrifuges. This magnetic-nanoparticle-based separation method significantly enhances the simplicity of our approach. Our CRISPR-based colorimetric biosensor offers a promising solution for early viral infection diagnosis, demonstrating the potential for integration into POCT systems.

## 2. Materials and Methods

### 2.1. Materials and Reagents

The ssDNA and target DNA were procured from Bioneer. The specific ssDNAs used were [Thiol]-5′-ATATATATATA-3′-[Biotin] and [Thiol]-5′-ATATATATATA-3′. For the targets, the following DNAs were utilized: HPV16L1: 5′-GTGCTGCCATATCTACTTCA-3′, HPV18L1: 5′-TGTGTAGAAGCACATATTGT-3′, HBV: 5′-TTGGCTTTCAGTTATATGGATGATGTGGTA-3′, and BRCA-1: 5′-GAACAAAAGGAAGAAAATCA-3′. The crRNA and Cas12a were obtained from IDT, with the crRNAs used being HPV16L1 crRNA: 5′-UAAUUUCUACUAAGUGUAGAUUGAAGUAGAUAUGGCAGCAC-3′ and HPV18L1 crRNA: 5′-UAAUUUCUACUCUUGUAGAUACAAUAUGUGCUUCUACACA-3′. Terminal transferase, streptavidin-HRP, dATP, and TMB solution were sourced from Sigma-Aldrich. Biotin-16-dUTP was obtained from Jena Bioscience. The sample was characterized using field emission scanning electron microscopy (FE-SEM; Gemini500, Zeiss, Jena, Germany) and Cs_corrected field emission transmission electron microscopy (FE-TEM; JEM-ARM200F, Jeol, Tokyo, Japan); the microscopes were installed in the Center for University-wide Research Facilities (CURF) at Jeonbuk National University. Fluorescence spectra and absorbance spectra were collected using a multi-mode microplate reader (Tecan, TECAN infinite 200 PRO, Männedorf, Switzerland).

### 2.2. Synthesis of 10 nm Fe_3_O_4_ Nanoparticles for Magnetic Core

The production of 10 nm Fe_3_O_4_ nanoparticles (MNP) involved several detailed steps. First, all glassware was dried and stored at 60 °C. Thereafter, a mixture of 1,2-hexadecanediol, oleylamine, oleic acid, and Fe(acac)_3_ was placed in a flask and heated to 130 °C for up to 90 min to evaporate residual water. The temperature was increased to 200 °C for two hours and then to 300 °C for an additional hour, followed by slow cooling to room temperature. The MNP solution was collected from the flask once the temperature was below 30 °C. Ethanol was added to the MNP solution and centrifuged to discard the supernatant. The remaining precipitate was re-dispersed in toluene and then centrifuged, and the supernatant was combined with ethanol and centrifuged again. The final supernatant was discarded and the precipitate re-dispersed in toluene, yielding the 10 nm MNP.

### 2.3. Synthesis of the AuNHs

The AuNHs were synthesized via a modified protocol [43,44]. Initially, 660 μL of CO-520 was dispersed in 12.6 mL of cyclohexane and stirred at 650 rpm for 30 min, after which 200 μL of a 1 mg MNP was added and stirred for another hour. Subsequently, 140 μL of NH_4_OH and 20 μL of tetraethylorthosilicate (TEOS) were added in sequence and stirred for 1 h and 48 h, respectively. The process continued with the addition of 3-aminopropyltriethoxysilane (APTES) and another 12 h of stirring at room temperature. The SiO_2_@Fe_3_O_4_ nanoparticles formed were purified by introducing 4.0 mL of a 50 mM tetramethylammonium hydroxide (TMAOH)/methanol solution, which was shaken briefly. The nanoparticles were collected using a magnet. After discarding the solvent, the nanoparticles were washed three times with a mixture of ethanol and deionized water (DIW) at 7500 rpm for 15 min and re-dispersed in 5 mL of DIW. A gold seed solution was prepared separately by mixing 0.5 mL of 1M NaOH with 38 mL of DIW and stirred for 3 min. Thereafter, 1 mL of 1% tetrakis(hydroxymethyl)phosphonium chloride (THPC) and 2 mL of 1% HauCl_4_ were added, and the mixture was stirred after each addition. For the formation of the gold shell, 30 μL of the Fe_3_O_4_–SiO_2_ solution was mixed with 1 mL of the gold seed solution and stirred for 30 min. After washing and centrifuging to remove excess gold seeds, the solution was re-dispersed in 1 mL of water. The final reaction involved the addition of 120 μL of a 0.5% polyvinylpyrrolidone (PVP) solution and 5 mL of K_2_CO_3_/HAuCl_4_ to the nanoparticle solution (with further stirring); the mixture was further stirred for 30 min after the addition of the prepared magnetic gold nanoparticle (MGNP) solution and 120 μL of 20 mM NH_2_OH HCl until a bluish-green color appeared, indicating the formation of gold nanoshells. The AuNHs were then washed with water to complete the process.

### 2.4. Elongation of ssDNA and Preparation of the AuNH–DNA–HRP Complex

A transferase reaction was conducted on an 11 bp ssDNA, which was thiolated only at the 5′ end. For the ssDNA, 1 mM of biotin-16-dUTP and 10 mM of dATP were used as nucleotides. This was mixed with terminal transferase, a TdT reaction buffer, and 25 mM CoCl_2_, and the reaction was conducted at 37 °C for 30 min. The ssDNA was then treated with DTT in preparation for its attachment to the AuNHs. The prepared AuNHs were washed three times with DIW before being combined with DTT-treated ssDNA and incubated at 37 °C for 3 h, resulting in DNA-coated AuNH. An overnight incubation was then performed using a blocking buffer composed of 3% BSA. This was followed by a 2 h incubation with streptavidin-HRP at room temperature. After three washing steps, the mixture was incubated at 37 °C for 2 h and then washed twice, yielding the prepared AuNH–DNA–HRP complex.

### 2.5. Detection of Viral DNAs Using the Activated CRISPR-Cas12a Complex on the AuNH Nanoparticles

The activated CRISPR-Cas12a complex was formed by reacting 60 nM Cas12a and 60 nM crRNA in RNase-free water for 10 min at 37 °C. Following the complex formation, the target DNA was introduced to the CRISPR-Cas12a complex in a cleavage buffer (20 mM HEPES, 150 mM KCl, 10 mM MgCl_2_, 1% glycerol, and 0.5 mM dithiothreitol). The activated solution was then incubated with AuNH–DNA–HRP at 37 °C for 2–3 h. Subsequently, the AuNHs were separated using a magnet for 5 min, and the supernatant underwent a TMB reaction for 30 min.

## 3. Results

### 3.1. Characterization of AuNH and Extension of ssDNA Using Terminal Transferase

**Figure 2:**
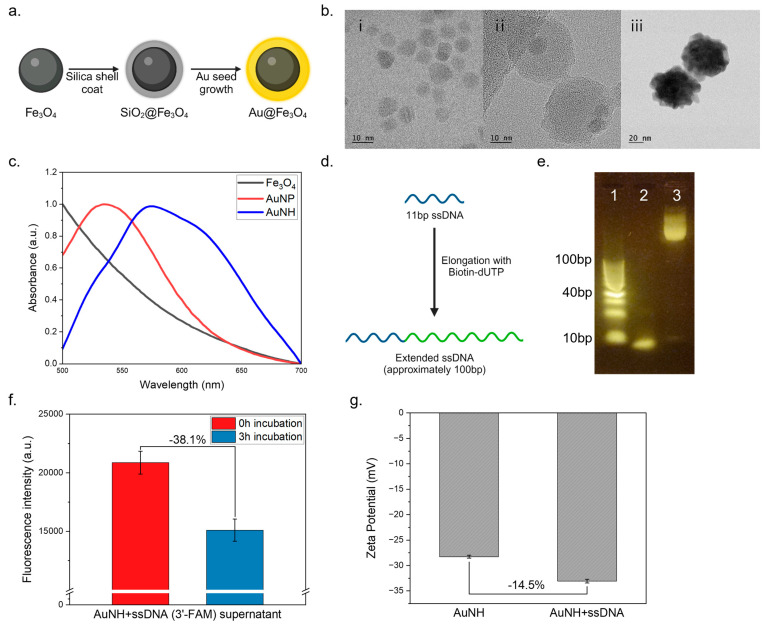
Characterization of particle and DNA elongation. (**a**) AuNH synthesis strategy; (**b**) TEM images of (**i**) Fe_3_O_4_, (**ii**) Fe_3_O_4_–SiO_2_, (**iii**) AuNH; (**c**) UV–vis absorbance spectrum of the AuNHs; (**d**) ssDNA elongation strategy; (**e**) ssDNA elongation electrophoresis conducted using a 4% agarose gel at 100 V for 40 min with a 10 bp ladder (**e1**), an ssDNA sample with the same concentration (**e2**), and the resulting transferase product (**e3**); (**f**) fluorescence observations of AuNH complexed with ssDNA and labeled with 3′-FAM following a 3 h incubation; (**g**) zeta potential of AuNH and ssDNA-coated AuNH.

The synthesis of hybrid nanoparticles with unique properties has attracted significant attention in various fields of research. AuNHs, which are composed of a magnetic Fe_3_O_4_ core and a gold Au shell, have garnered interest because of their potential application in drug delivery and biosensing. First, we synthesized AuNH nanoparticles using the seed growth method, focusing on the advantages of the Fe_3_O_4_ core for easy magnetic separation and the Au shell for easier DNA attachment for further functionalization. The AuNH nanoparticles were synthesized using the seed growth method, as depicted in Figure 2a. Initially, Fe_3_O_4_ nanoparticles were synthesized using a chemical precipitation method. Subsequently, the Fe_3_O_4_ nanoparticles were used as seeds for growing the Au shell via a reduction reaction. In Figure 2(bi), the TEM image of Fe_3_O_4_ is presented. Subsequent TEM analysis in Figure 2(bii) reveals the presence of the Fe_3_O_4_–SiO_2_ silica shell, demonstrating the successful formation of a core–shell structure. In Figure 2(biii), the TEM analysis confirms that the Fe_3_O_4_ core is encapsulated within the Au shell. This observation demonstrates the successful synthesis of the AuNH core–shell nanostructure. The absorption spectra exhibited a peak at 590 nm indicative of the plasmon resonance of the Au nanoparticles (Figure 2c), further validating the synthesis of the AuNHs.

For the ssDNA extension, an 11 bp ssDNA was elongated using dATP and biotin-dUTP as nucleotides (Figure 2d). Agarose gel electrophoresis of the extended DNA products showed distinct bands corresponding to the normal DNA and the extended DNA (Figure 2e). The extended DNA fragments were observed to be over 100 bp long, indicating successful extension. The presence of relatively long DNA fragments suggests the incorporation of multiple biotin moieties, which can enhance the binding capacity of streptavidin-HRP for further functionalization. To ensure the successful attachment of ssDNA to the AuNH nanoparticles, we conducted a preliminary investigation using fluorescently labeled ssDNA (3′-FAM). Incubation was carried out for 3 h (Figure 2f), after which the magnetic separation of the nanoparticles enabled the isolation of the AuNH–ssDNA complex, while any unbound ssDNA remained in the supernatant. By measuring the fluorescence intensity of the supernatant after incubation, we determined the extent to which ssDNA had bonded to the AuNH nanoparticles. As a result, the fluorescence intensity in the supernatant decreased substantially after 3 h of incubation, indicating the successful binding of ssDNA to the AuNH nanoparticles. Additionally, we measured the zeta potential of both AuNH and ssDNA-coated AuNHs. The AuNHs were incubated with ssDNA for 3 h, followed by two washing steps, after which the zeta potential was assessed. The results indicated that the ssDNA-coated AuNHs exhibited an increased negative charge, confirming the successful coating of ssDNA onto the AuNH surface (Figure 2g).

### 3.2. Signal Amplification in CRISPR-Based DNA Detection Using Extended DNA on the AuNH Nanoparticles

**Figure 3:**
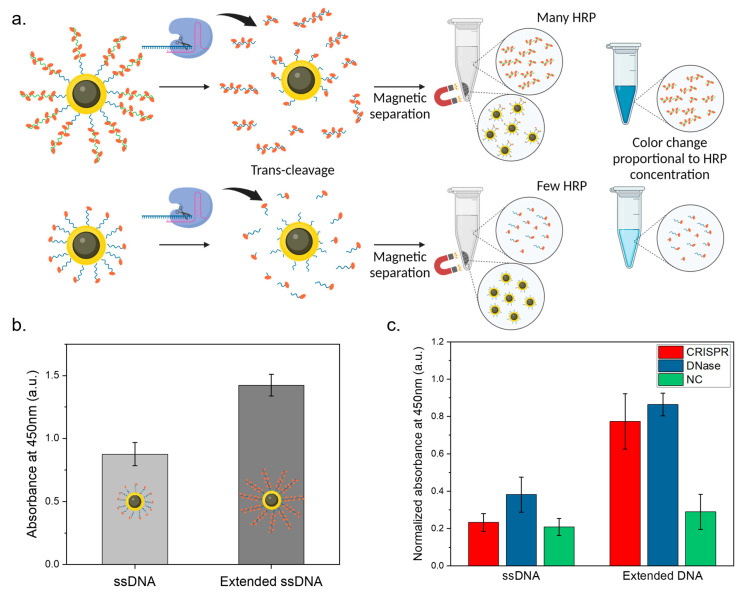
Signal amplification in CRISPR-based DNA detection. (**a**) Strategy for signal enhancement via HRP; (**b**) comparison of absorbance at 450 nm between AuNH–DNA–HRP complexes and either normal ssDNA or elongated ssDNA; (**c**) absorbance comparison between normal ssDNA and elongated ssDNA at 450 nm from the CRISPR reaction supernatant.

Signal amplification is crucial in achieving heightened sensitivity in DNA detection assays. In our study, we employed terminal transferase and biotin-dUTP to extend an 11 bp ssDNA; this resulted in a relatively long DNA strand with multiple streptavidin-HRP binding sites. This signal amplification strategy was compared to the use of normal 11 bp DNA (3′-biotin), with the expectation that the extended DNA strand would yield a stronger signal owing to the increased number of streptavidin-HRP binding sites (Figure 3a).

Next, to validate the signal amplification in the CRISPR-based DNA detection, we compared the AuNHs functionalized with 11 bp ssDNA and extended DNA. The design of the extended DNA strands with multiple streptavidin-HRP binding sites could enhance signal intensity more efficiently than the normal 11 bp DNA, which features only a single streptavidin-HRP binding site. The AuNHs were first conjugated with the respective DNA and streptavidin-HRP. After thorough washing to eliminate unbound molecules, we obtained the AuNH–ssDNA–HRP complex. Before the CRISPR reaction, the AuNHs were separately functionalized with normal DNA and extended DNA. The AuNH–ssDNA–HRP complexes were then prepared and subjected to a TMB reaction. In Figure 3b, the extended DNA could incorporate more HRP than normal DNA, even at the same AuNH concentration. As a result, a stronger signal was observed from AuNH–ssDNA–HRP complexes, particularly from the extended ssDNA-attached AuNHs, resulting in a 38.5% higher signal compared to other particles. Subsequently, we assessed the signal amplification efficiency using CRISPR Cas12a trans-cleavage activity. The enzyme cleaved the ssDNA attached to the AuNH, and the nanoparticles were efficiently and easily separated using a magnet, leaving the broken DNA strands and the HRP bound to them in the supernatant. This supernatant was subjected to a TMB reaction to quantify the signal generated from the broken DNA strands. The analysis of the TMB reaction absorbance peaks after CRISPR reaction revealed that the 11 bp normal DNA exhibited minimal differentiation from the negative control at a target DNA concentration of 20 nM. In contrast, the extended DNA displayed a distinct and significant signal increase as compared to the negative control at the same concentration. Concretely, in Figure 3c, following the nucleic acid cleavage reaction of CRISPR and DNase, the supernatant exhibited a stronger signal from the extended ssDNA-attached AuNHs. Additionally, the extended DNA displayed a distinct and significant signal increase compared to the negative control. These compelling findings provide substantial evidence for the successful enhancement of signal amplification for the sensitive detection of CRISPR targets even at low concentrations, demonstrating the effectiveness of our signal amplification strategy.

### 3.3. Specific DNA Target Detection Using a CRISPR-Based Biosensor System with Enhanced Signal Amplification

In this study, we verified the ability of a CRISPR-based biosensor system to detect specific DNA targets. The system utilizes CRISPR-Cas12a ribonucleoprotein (RNP) complexed with HPV16L1 crRNA to allow for the selective detection of the corresponding DNA targets.

**Figure 4:**
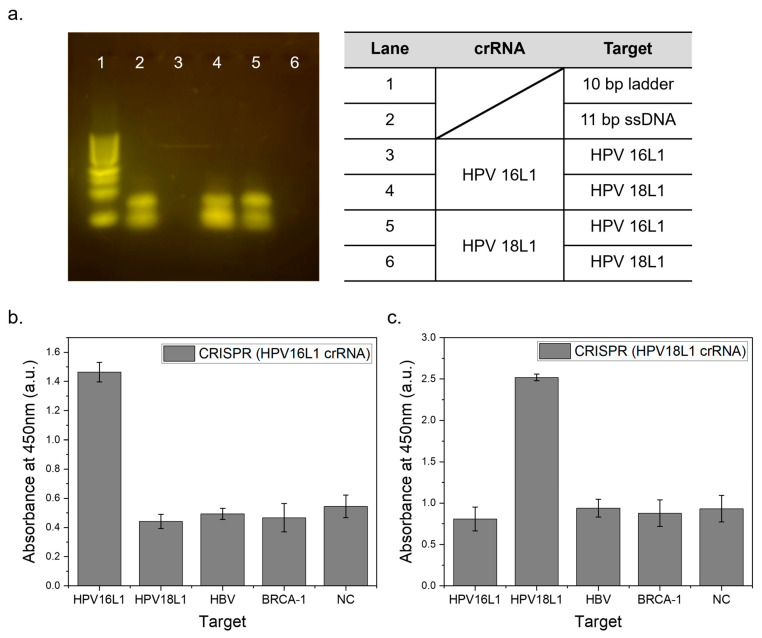
Specific target detection according to crRNA sequences. (**a**) Electrophoresis results following the CRISPR reaction, with a 10 bp ladder (1), ssDNA sample with the same concentration (2), HPV16L1 crRNA targeting HPV16L1 (3), and HPV18L1 (4) and HPV18L1 crRNA targeting HPV16L1 (5) and HPV18L1, respectively (6); (**b**) absorbance at 450 nm of the supernatant from the CRISPR reaction using HPV16L1 crRNA against HPV16L1, HPV18L1, HBV, and BRCA-1 targets and negative control, all tested with extended ssDNA; (**c**) similarly measured absorbance for products using HPV18L1 crRNA.

Before applying this system, we first confirmed the specificity using gel electrophoresis, where trans-cleavage reactions occurred only when the crRNA and target DNA were complementary. As shown in Figure 4a, when the target DNA matched HPV16L1 crRNA, only enzymatic reactions occurred, and distinct gel electrophoresis bands were not visible (lane 3). Conversely, when HPV18L1 crRNA was used, enzymatic reactions only occurred with target DNA matching HPV18L1, as evidenced by specific bands on the gel electrophoresis (lane 6). Only the control group with ssDNA, represented in lane 2, as well as lane 4 and 5 wherein no CRISPR reaction occurred, exhibited a 20 bp band, which is attributed to the strong SH–SH bonding of 5′ Thiol DNA.

This system was expected to react selectively with specific DNA targets because of the complementary binding between crRNA and the target DNA. Figure 4b shows the absorbance peak at 450 nm of the TMB reaction results in the CRISPR-based biosensor system for HPV16L1 targets. The presence of a complementary HPV16L1 crRNA-matched HPV16L1 DNA target produced a definite signal, indicating that Cas12a had completed the trans-cleavage. In contrast, no significant reaction occurred with HPV18L1, HBV, and BRCA-1 targets; as a result, signal levels similar to those of the negative control were observed. This selective response further confirms the specificity of the biosensor system, with only the HPV16L1 target eliciting a distinct and reliable signal, while other noncomplementary targets showed minimal signal intensity, akin to the negative control. Figure 4c shows the test conducted using HPV18L1 crRNA; the HPV 18L1 DNA target produced a distinct signal. In contrast, neither the noncomplementary DNA targets nor the negative control showed any significant reaction. Additionally, the signal intensity was enhanced by increasing the HRP attachment. The selective binding between specific crRNA and its corresponding target DNA ensured accurate detection and a distinct differentiation from nontarget DNA. The successful validation of this system with HPV16L1 and HPV18L1 targets highlights its potential for detecting other virus DNA targets in future tests.

### 3.4. Sensitivity Validation and Target Detection in a CRISPR-Based Biosensor System for DNA Virus Detection

To evaluate the sensitivity and capabilities of our CRISPR-based biosensor system for DNA virus detection, we conducted tests using CRISPR Cas12a and HPV16L1 crRNA. These tests were specifically designed to detect HPV16L1 targets, both in isolation and when mixed in human serum, to demonstrate the effectiveness of the biosensor in different scenarios.

**Figure 5:**
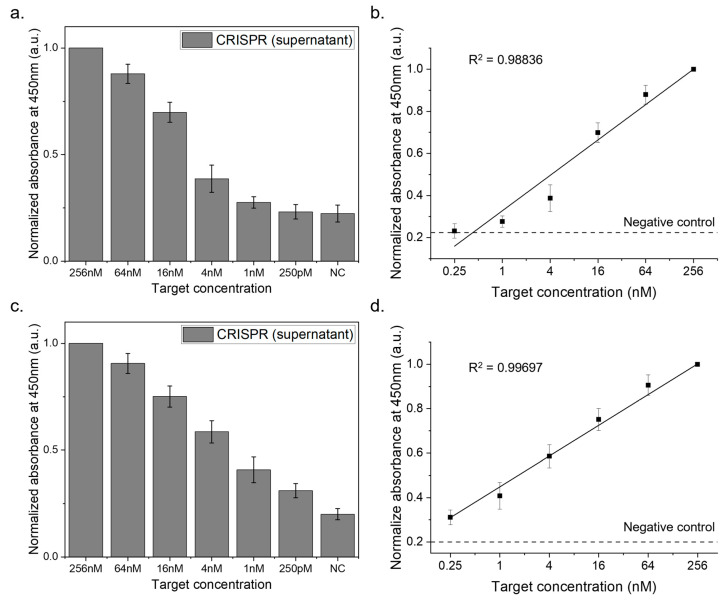
Sensitivity analysis of the CRISPR-based biosensor system. This figure displays the outcomes of the sensitivity tests performed with the CRISPR biosensor, where HPV16L1 was targeted in two distinct setups. First, the tests were conducted with standard HPV16L1 targets and subsequently conducted with HPV16L1 targets mixed in human serum. After the CRISPR reaction in each scenario, the supernatant was separated, and its absorbance at 450 nm was assessed after a TMB reaction. (**a**) Detection of HPV16L1 targets across a range of 256 nM to 250 pM. (**b**) Linear curve with an LOD of 0.25 nM. (**c**) Detection of HPV16L1 targets in human serum across a range of 256 nM to 250 pM. (**d**) Linear curve with an LOD of 0.25 nM in human serum context.

The sensitivity tests covered a total target concentration range of 256 nM to 250 pM. Figure 5a shows the results for the HPV16L1 target detection using HPV16L1 crRNA. As the total target concentration decreased from 256 nM to 250 pM, the biosensor system successfully detected the targets with reliable signals. Figure 5b illustrates linear curves plotted for these tests, demonstrating the method accuracy with an R^2^ value of 0.98 and a limit of detection (LOD) of 0.25 nM.

To simulate real sample conditions, we diluted the target DNA in human serum with PBS at a 1/20 ratio. The results, as shown in Figure 5c, indicate that the biosensor effectively identified HPV16L1 targets even amidst human serum, underscoring its practical applicability. The corresponding linear curves (Figure 5d) achieved an R^2^ value of 0.99, reaffirming the precision and accuracy of the method, with an LOD of 0.25 nM in the presence of human serum.

## 4. Discussion

In this study, we successfully developed a CRISPR-based biosensor system for the sensitive and specific detection of DNA virus targets (HPV16L1 and HPV18L1). We synthesized AuNH with a core–shell structure, which served as an excellent platform for functionalization and easy magnetic separation. By extending the ssDNA using terminal transferase and biotin-dUTP, we achieved signal amplification through the increased binding capacity of streptavidin-HRP. This signal amplification strategy achieved an LOD as low as 0.25 nM, offering enhanced sensitivity for DNA virus detection. Through specificity validations, we confirmed that the CRISPR-based biosensor system selectively detected HPV16L1 and HPV18L1 targets with distinct and specific signals. Moreover, we demonstrated the adaptability of the system in detecting other DNA virus targets by modifying the crRNA sequences; the sensitivity and detection efficiency were still maintained. Overall, the CRISPR-based biosensor system in this study holds promise for practical applications in DNA virus detection, including early diagnostics and surveillance. The high sensitivity, specificity, and versatility of the system highlight its potential as a valuable tool in the field of viral detection. This system can contribute to the effective management and control of viral infections. However, it is worth noting that, during the sensitivity test, the absorbance tested with human serum appeared to be lower overall compared to the pure target. This discrepancy is likely attributed to the complex sample matrix of human serum [45], which may have hindered the enzyme reactions in our system. Moreover, the overall process from CRISPR reaction to TMB detection took a longer time, which is 3 h. Hence, in order to be implemented in a POCT system, reduction in detection time is deemed to be crucial. This limitation will be further studied and addressed in the next experiment in the near future. Future applications may involve the detection of various DNA virus targets and the development of point-of-care diagnostic devices for rapid and accurate virus detection.

## 5. Conclusions

The developed CRISPR-Cas12a-based biosensor system in this study shows significant potential for enhancing the detection of DNA viruses, particularly HPV16L1. The key to its effectiveness lies in the novel signal amplification strategy that uses extended ssDNA to increase sensitivity. This system can detect HPV16L1 at very low concentrations, demonstrating a substantial improvement over the current diagnostic techniques and highlighting its potential for early and accurate disease detection. Notably, this system is applicable in POCT, making it advantageous to resource-limited environments. The simplicity and quick response of the system are crucial for effective viral detection in diseases and outbreak control. However, our study recognizes the challenges in optimizing the sensitivity of the assay and emphasizes the need for additional research to enhance its sensitivity in detecting targets, which is crucial for its widespread application in POCT without compromising specificity. Since HPV is a global problem and is linked to cervical cancer, the ability of our biosensor to accurately detect high-risk DNA viruses is significant for public health. It holds tremendous potential in improving disease surveillance, aiding early diagnosis, and contributing to global disease prevention and control, making it a noteworthy advancement in viral diagnostics and public health.

## Data Availability

Data are contained within the article.

## References

[1] Bray F., Ferlay J., Soerjomataram I., Siegel R.L., Torre L.A., Jemal A. (2018). Global cancer statistics 2018: GLOBOCAN estimates of incidence and mortality worldwide for 36 cancers in 185 countries. CA A Cancer J. Clin..

[2] de Martel C., Georges D., Bray F., Ferlay J., Clifford G.M. (2020). Global burden of cancer attributable to infections in 2018: A worldwide incidence analysis. Lancet Glob. Health.

[3] Lyu Z., Feng X., Li N., Zhao W., Wei L., Chen Y., Yang W., Ma H., Yao B., Zhang K. (2017). Human papillomavirus in semen and the risk for male infertility: A systematic review and meta-analysis. BMC Infect. Dis..

[4] Chabeda A., Yanez R.J., Lamprecht R., Meyers A.E., Rybicki E.P., Hitzeroth I.I. (2018). Therapeutic vaccines for high-risk HPV-associated diseases. Papillomavirus Res..

[5] Chrysostomou A.C., Stylianou D.C., Constantinidou A., Kostrikis L.G. (2018). Cervical cancer screening programs in Europe: The transition towards HPV vaccination and population-based HPV testing. Viruses.

[6] Tommasino M. (2014). The human papillomavirus family and its role in carcinogenesis. Semin. Cancer Biol..

[7] Berman T.A., Schiller J.T. (2017). Human papillomavirus in cervical cancer and oropharyngeal cancer: One cause, two diseases. Cancer.

[8] Bhatla N., Singhal S. (2020). Primary HPV screening for cervical cancer. Best Pract. Res. Clin. Obstet. Gynaecol..

[9] Halec G., Alemany L., Lloveras B., Schmitt M., Alejo M., Bosch F.X., Tous S., Klaustermeier J.E., Guimerà N., Grabe N. (2014). Pathogenic role of the eight probably/possibly carcinogenic HPV types 26, 53, 66, 67, 68, 70, 73 and 82 in cervical cancer. J. Pathol..

[10] Xi L.F., Schiffman M., Koutsky L.A., Hughes J.P., Hulbert A., Shen Z., Galloway D.A., Kiviat N.B. (2016). Variant-specific persistence of infections with human papillomavirus Types 31, 33, 45, 56 and 58 and risk of cervical intraepithelial neoplasia. Int. J. Cancer.

[11] Hadi A., Al-Mawlah Y., Al-Janabi W., Majeed L. (2023). Human Papillomavirus: It’s Characteristics, Pathogenesis, Transmission, Immunity, and it’s Role in Cervical Cancer: A Mini Review. J. Water Res..

[12] Brisson M., Drolet M. (2019). Global elimination of cervical cancer as a public health problem. Lancet Oncol..

[13] Canfell K., Kim J.J., Brisson M., Keane A., Simms K.T., Caruana M., Burger E.A., Martin D., Nguyen D.T., Bénard É. (2020). Mortality impact of achieving WHO cervical cancer elimination targets: A comparative modelling analysis in 78 low-income and lower-middle-income countries. Lancet.

[14] Brisson M., Kim J.J., Canfell K., Drolet M., Gingras G., Burger E.A., Martin D., Simms K.T., Bénard É., Boily M.-C. (2020). Impact of HPV vaccination and cervical screening on cervical cancer elimination: A comparative modelling analysis in 78 low-income and lower-middle-income countries. Lancet.

[15] Bhatla N., Singla S., Awasthi D. (2012). Human papillomavirus deoxyribonucleic acid testing in developed countries. Best Pract. Res. Clin. Obstet. Gynaecol..

[16] Tota J.E., Bentley J., Blake J., Coutlée F., Duggan M.A., Ferenczy A., Franco E.L., Fung-Kee-Fung M., Gotlieb W., Mayrand M.-H. (2017). Introduction of molecular HPV testing as the primary technology in cervical cancer screening: Acting on evidence to change the current paradigm. Prev. Med..

[17] Mandal R., Basu P. (2018). Cancer screening and early diagnosis in low and middle income countries. Bundesgesundheitsbl.

[18] Rajaram S., Gupta B. (2021). Screening for cervical cancer: Choices & dilemmas. Indian J. Med. Res..

[19] Campos N.G., Tsu V., Jeronimo J., Mvundura M., Kim J.J. (2017). Estimating the value of point-of-care HPV testing in three low-and middle-income countries: A modeling study. BMC Cancer.

[20] Vallely A.J., Saville M., Badman S.G., Gabuzzi J., Bolnga J., Mola G.D., Kuk J., Wai M., Munnull G., Garland S.M. (2022). Point-of-care HPV DNA testing of self-collected specimens and same-day thermal ablation for the early detection and treatment of cervical pre-cancer in women in Papua New Guinea: A prospective, single-arm intervention trial (HPV-STAT). Lancet Glob. Health.

[21] Toliman P.J., Kaldor J.M., Badman S.G., Gabuzzi J., Silim S., Kumbia A., Kombuk B., Kombati Z., Munnull G., Guy R. (2018). Performance of clinical screening algorithms comprising point-of-care HPV-DNA testing using self-collected vaginal specimens, and visual inspection of the cervix with acetic acid, for the detection of underlying high-grade squamous intraepithelial lesions in Papua New Guinea. Papillomavirus Res..

[22] Jung W., Han J., Choi J.-W., Ahn C.H. (2015). Point-of-care testing (POCT) diagnostic systems using microfluidic lab-on-a-chip technologies. Microelectron. Eng..

[23] Luppa P.B., Müller C., Schlichtiger A., Schlebusch H. (2011). Point-of-care testing (POCT): Current techniques and future perspectives. TrAC Trends Anal. Chem..

[24] Meagher R.J., Hatch A.V., Renzi R.F., Singh A.K. (2008). An integrated microfluidic platform for sensitive and rapid detection of biological toxins. Lab Chip.

[25] Carneiro M.C., Rodrigues L.R., Moreira F.T., Sales M.G.F. (2022). based ELISA for fast CA 15–3 detection in point-of-care. Microchem. J..

[26] Beall M.J., Mainville C.A., Arguello-Marin A., Clark G., Lemieux C., Saucier J., Thatcher B., Breitschwerdt E.B., Cohn L.A., Qurollo B.A. (2022). An improved point-of-care ELISA for the diagnosis of anaplasmosis and ehrlichiosis during the acute phase of tick-borne infections in dogs. Top. Companion Anim. Med..

[27] Gupta N., Augustine S., Narayan T., O’Riordan A., Das A., Kumar D., Luong J.H., Malhotra B.D. (2021). Point-of-care PCR assays for COVID-19 detection. Biosensors.

[28] Petralia S., Conoci S. (2017). PCR technologies for point of care testing: Progress and perspectives. ACS Sens..

[29] Joung J., Ladha A., Saito M., Kim N.-G., Woolley A.E., Segel M., Barretto R.P., Ranu A., Macrae R.K., Faure G. (2020). Detection of SARS-CoV-2 with SHERLOCK one-pot testing. N. Engl. J. Med..

[30] Aman R., Marsic T., Sivakrishna Rao G., Mahas A., Ali Z., Alsanea M., Al-Qahtani A., Alhamlan F., Mahfouz M. (2022). iSCAN-V2: A one-pot RT-RPA–CRISPR/Cas12b assay for point-of-care SARS-CoV-2 detection. Front. Bioeng. Biotechnol..

[31] Choi J.-H., Lim J., Shin M., Paek S.-H., Choi J.-W. (2020). CRISPR-Cas12a-based nucleic acid amplification-free DNA biosensor via Au nanoparticle-assisted metal-enhanced fluorescence and colorimetric analysis. Nano Lett..

[32] Faure G., Shmakov S.A., Yan W.X., Cheng D.R., Scott D.A., Peters J.E., Makarova K.S., Koonin E.V. (2019). CRISPR–Cas in mobile genetic elements: Counter-defence and beyond. Nat. Rev. Microbiol..

[33] McCarty N.S., Graham A.E., Studená L., Ledesma-Amaro R. (2020). Multiplexed CRISPR technologies for gene editing and transcriptional regulation. Nat. Commun..

[34] Yan W.X., Hunnewell P., Alfonse L.E., Carte J.M., Keston-Smith E., Sothiselvam S., Garrity A.J., Chong S., Makarova K.S., Koonin E.V. (2019). Functionally diverse type V CRISPR-Cas systems. Science.

[35] Li S.-Y., Cheng Q.-X., Wang J.-M., Li X.-Y., Zhang Z.-L., Gao S., Cao R.-B., Zhao G.-P., Wang J. (2018). CRISPR-Cas12a-assisted nucleic acid detection. Cell Discov..

[36] Pickar-Oliver A., Gersbach C.A. (2019). The next generation of CRISPR–Cas technologies and applications. Nat. Rev. Mol. Cell Biol..

[37] Mukama O., Wu J., Li Z., Liang Q., Yi Z., Lu X., Liu Y., Liu Y., Hussain M., Makafe G.G. (2020). An ultrasensitive and specific point-of-care CRISPR/Cas12 based lateral flow biosensor for the rapid detection of nucleic acids. Biosens. Bioelectron..

[38] Wang B., Wang R., Wang D., Wu J., Li J., Wang J., Liu H., Wang Y. (2019). Cas12aVDet: A CRISPR/Cas12a-based platform for rapid and visual nucleic acid detection. Anal. Chem..

[39] Wu H., Qian C., Wu C., Wang Z., Wang D., Ye Z., Ping J., Wu J., Ji F. (2020). End-point dual specific detection of nucleic acids using CRISPR/Cas12a based portable biosensor. Biosens. Bioelectron..

[40] You S.-M., Luo K., Jung J.-Y., Jeong K.-B., Lee E.-S., Oh M.-H., Kim Y.-R. (2020). Gold nanoparticle-coated starch magnetic beads for the separation, concentration, and SERS-based detection of E. coli O157: H7. ACS Appl. Mater. Interfaces.

[41] Dong J., Yang H., Zhao J., Wen L., He C., Hu Z., Li J., Huo D., Hou C. (2022). Sandwich-type microRNA biosensor based on graphene oxide incorporated 3D-flower-like MoS2 and AuNPs coupling with HRP enzyme signal amplification. Microchim. Acta.

[42] Kim H.-S., Oh B.-K. (2014). A rapid and sensitive immunoassay for detection of *E. coli* O157: H7 using multienzyme—Au nanoparticle complex. BioChip J..

[43] Ding H.L., Zhang Y.X., Wang S., Xu J.M., Xu S.C., Li G.H. (2012). Fe_3_O_4_@SiO_2_ Core/Shell Nanoparticles: The Silica Coating Regulations with a Single Core for Different Core Sizes and Shell Thicknesses. Chem. Mater..

[44] Kang M., Kim Y. (2020). Au-coated Fe_3_O_4_@SiO_2_ core-shell particles with photothermal activity. Colloids Surf. A Physicochem. Eng. Asp..

[45] Hu Y., Liu L., Wang C., Zhou J., Liu R., Lv Y. (2024). CRISPR-Cas12a-Enhanced Mass Spectrometric DNA Nanomachine for HPV-16 Detection in Human Serum. Chem. Commun..

